# Tailored synbiotic powder (functional food) to prevent hyperphosphataemia (kidney disorder)

**DOI:** 10.1038/s41598-021-95176-3

**Published:** 2021-08-13

**Authors:** Ajeeta Anand, Shigeki Yoshida, Hideki Aoyagi

**Affiliations:** Faculty of Life and Environmental Sciences, University of Tsukuba, Tsukuba, Ibaraki 305-8572 Japan

**Keywords:** Biological techniques, Microbiology, Health care, Urology

## Abstract

Hyperphosphataemia is treated with phosphate binders, which can cause adverse effects. Spray-dried synbiotic powder (SP) composed of *Lactobacillus casei* JCM1134 (a phosphate-accumulating organism; PAO) and *Aloe vera* is potentially a safer alternative for efficient phosphate removal. In this study, a novel strategy was developed; lysine-derivatized deacetylated *A. vera* (DAVK) was synthesised and fabricated on phosphate-deficient PAO (PDP) for efficient phosphate transfer and then spray-dried with the supernatant of DAV centrifugation to form a sacrificial layer on PDP for SP integrity during gastric passage. In vitro experiments revealed that PAO removed only 1.6% of the phosphate from synthetic media, whereas SP removed 89%, 87%, and 67% (w/v) of the phosphate from milk, soft drink, and synthetic media, respectively, confirming the protective role of *A. vera* and efficient phosphate transport. Compared with commercial binders, SP effectively removed phosphate from synthetic media, whereas SP and CaCO_3_ exhibited comparative results for milk and soft drink. Importantly, CaCO_3_ caused hypercalcaemia. Thus, the described SP presents a promising tool to prevent hyperphosphataemia. This study also revealed a novel factor: diets of patients with chronic kidney disease should be monitored to determine the optimal phosphate binders, as phosphate removal performance depends on the accessible phosphate forms.

## Introduction

Hyperphosphataemia occurs when there is a positive phosphate balance in the blood induced by reduced renal function in chronic kidney disease (CKD)^1,2^. The prevalence of hyperphosphataemia is approximately 50$\sim$74% among patients with kidney disease^3^, which was associated with approximately 11$\sim$14% mortality in Brazil in 2014^4^. The occurrence of CKD is most frequent among the older people in developed and developing countries, where older people are or will be the majority of the population^5^. The prevention of hyperphosphatemia requires immediate attention since its onset is the driver of several life-threatening conditions such as vascular calcification-induced heart diseases, bone disorders or fractures caused by bone-mineral imbalance, and metabolic acidosis^6^.

Dialysis and chemical-based phosphate binders are common therapies used to remove excessive phosphate from the blood and restrict its assimilation into the body, respectively. However, recent reports demonstrate that phosphate binders are associated with various adverse effects including gastrointestinal disorders and heavy metal deposition, which requires long-term phosphate removal treatment with protein-restricted diet^7,8^ that may cause Kwashiorkor and Marasmus malnutrition. Calcium-based phosphate binders are the first line of treatment for hyperphosphatemia, but recent reports confirmed that they cannot be prescribed to patients since they may lead to hypercalcaemia and further worsen the CKD-related adversities, such as vascular calcification and bone-mineral imbalance^9^. Aluminium- and lanthanum-based binders were introduced but have limited applications as they can be deposited in bones and cause heavy metal toxicities^10,11^. Recently, sevelamer and ferro sulphoxide were established as novel phosphate binders; nonetheless, they were also reported to be associated with low fat-soluble vitamin bioavailability and diarrhoea, respectively^12,13^. These treatment strategies have high pill burden and cost for patients^14^, and are prescribed following CKD onset for hyperphosphataemia management when the damages to the kidney are difficult to overcome and the patients have poor quality of life^15^. These reports marked the clear research thrust to develop a novel and healthier strategy to prevent hyperphosphatemia at early stages.

In a previous investigation, we screened phosphate-accumulating organisms (PAOs) and identified bifidobacteria and lactic acid bacteria has holding great potential^16^. To obtain the best results for phosphate removal and compete with phosphate binders, *Lactobacillus casei* JCM1134 was isolated as an elite PAO^17^. However, to accurately estimate the phosphate removal by the elite PAO, we developed an accurate and reliable microbial phosphate estimation method^18^. To safely deliver the elite PAO with its bio-functionality at the duodenum, where phosphate assimilation begins and mainly occurs, followed by jejunum and then ileum^19^, we designed a synbiotic formulation by encapsulating (natural microbial-substratum attachment) *L. casei* JCM1134 (PAO, a probiotic) using chemically modified *Aloe vera* (prebiotic), which was made partially specific for the phosphate transfer and to serve as the sacrificial layer in the gut. To achieve these objectives, a stable and bio-functional spray-dried synbiotic formulation (SP, a functional food) was developed using novel strategies.

Various studies have focused on microbial encapsulation with materials using natural microbial attachment processes. A multi-step attachment process includes conditioning the substratum with organic matter to supply nutrients^20,21^ and microbial transport to the substratum surface through fluid flow or intrinsic microbial mobility^22,23^. The attachment between conditioned films and microbes depends on molecular forces involving Lifshitz-van der Waals forces, electrostatic, and acid–base interactions^24,25^.

Recently, *A. vera* was reported as an encapsulating agent for the stability and bio-functionality of biomolecules during spray drying^26^. The incorporation of *A. vera* in commercialised functional foods has attracted significant attention^27,28^. *A. vera* has various beneficial effects on wound healing, intestinal disorders, and cancer^29^. In this study, *A. vera* was selected due to its low phosphate content (⁓0.7% w/w) and its acemannan, a well-known prebiotic^30^ which makes *A. vera* suitable and safe for phosphate removal and stability of SP in vitro.

**Figure 1:**
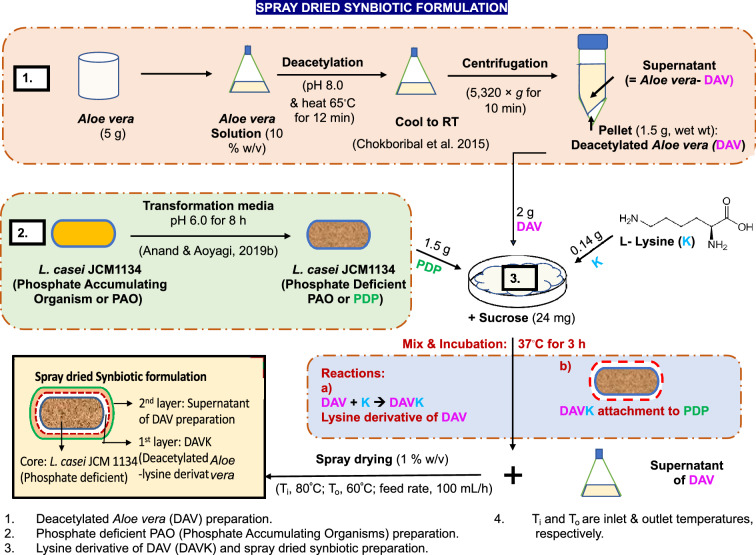
Experimental procedure for spray dried synbiotic powder (SP) formulation.

In this study, PAO was transformed into phosphate deficient PAO (PDP) to increase its phosphate accumulation capacity. However, the encapsulating agent, *A. vera*, was separated into two parts via a deacetylation reaction: (1) deacetylated *A. vera* (DAV), further modified with lysine (K), producing K-derivatised DAV (DAVK, which is insoluble and has an affinity for phosphate), and used to construct the first encapsulation layer to facilitate efficient phosphate accumulation and maintaining SP integrity. (2) The supernatant of the DAV centrifugation was then used to prepare the sacrificial (second) layer by spray drying to maintain SP integrity in the simulated gastric conditions. The double-encapsulated SP was evaluated for its phosphate removal ability in vitro and compared with commercially available phosphate binders. A brief illustration of the methodology and terminology are shown in Fig. 1.

## Results

### DAV preparation and characterisation

**Figure 2:**
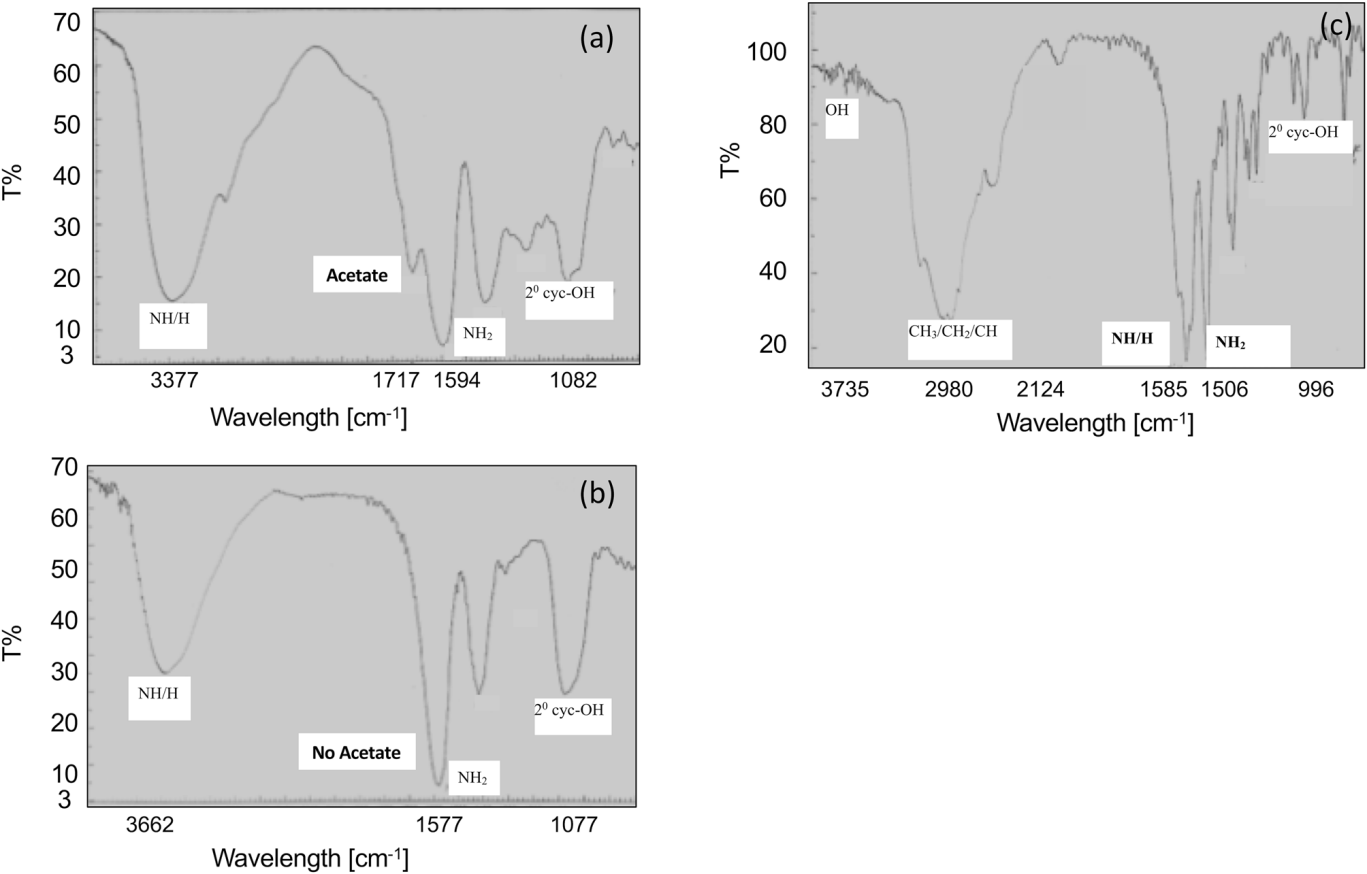
Fourier-transform infrared spectroscopy (FT-IR) analysis of *Aloe vera* modification. (**a**) Untreated *Aloe vera*, (**b**) deacetylated *A. vera* (DAV), and (**c**) lysine derivative of DAV (DAVK).

Following *A. vera* deacetylation, 30% (wet w/w) DAV was obtained with a purity of 98.6% (w/w) by analysing the sugar-rich content; the deacetylation of *A. vera* was confirmed using Fourier-transform infrared spectroscopy (FT-IR) analysis. The peak of the acetate group in the untreated *A. vera* (Fig. 2a) was completely absent from the deacetylated *A. vera* (Fig. 2b), as there was no transmittance peak observed for acetate at 1,800$\sim$1,700 cm^−1^, which confirmed nearly complete *A. vera* deacetylation. The composition of *A. vera* is summarised in Supplementary Table S1 and its sugar analysis was conducted using high-performance liquid chromatography and gas chromatography (GC) techniques. The sugar constituents of *A. vera* (Supplementary Table S2), comprised glucose and mannose in a 2:1 ratio, as shown in GC analysis (Supplementary Fig. S1). Therefore, the carbohydrate used in the present study was glucomannan (1.1324 KDa) with 6$\sim$7 monomers (Supplementary Fig. S2). We also found that the sugar content in *A. vera* depended on the hydrolysis conditions and sample preparation.

### Lysine derivatisation of DAV and its encapsulation on PDP

**Figure 3:**
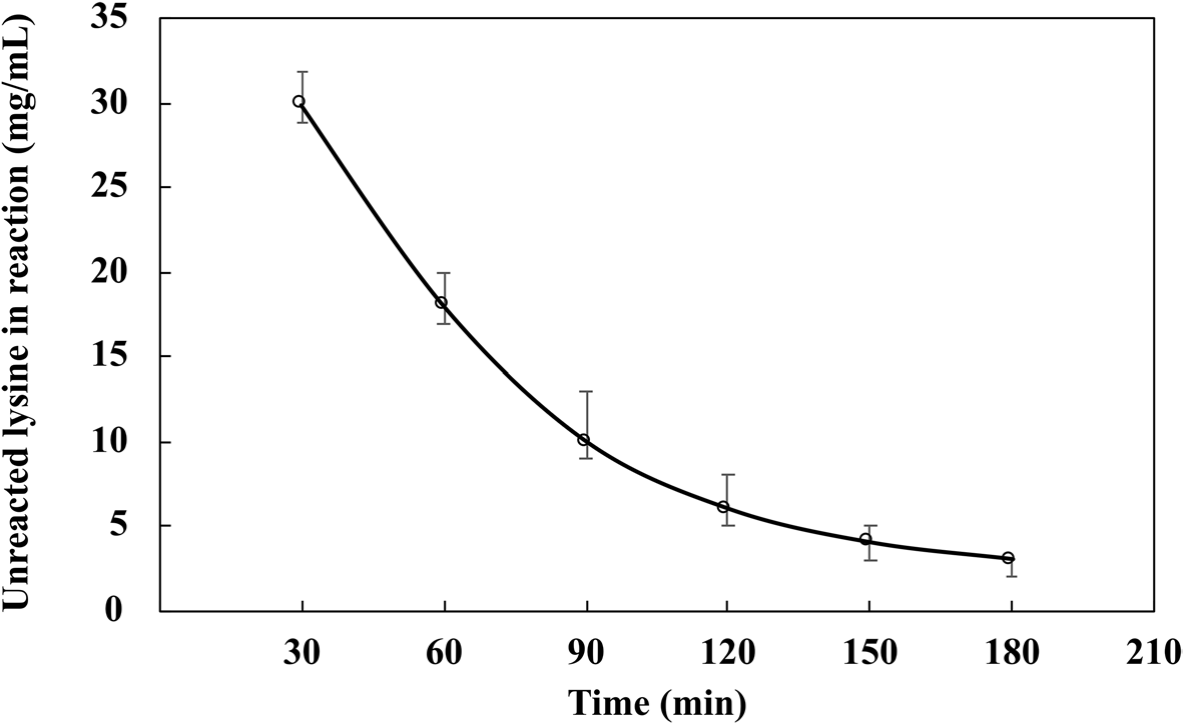
Unreacted lysine in lysine derivative of deacylatisation *Aloe vera* (DAVK) over time. In the reaction mixture, deacylated *A. vera* (DAV, 1 g) and lysine (70 mg) reacted under optimised conditions and significant *p*-values (*p* < 0.002 by two-tailed ANOVA, with n = 3 and alpha value of 0.95) were obtained and the standard errors are shown as error bars.

Lysine derivatisation of DAV was essential to reduce the zeta potential (− 39.17 mV) to obtain the favourable conditions for phosphate interaction. For successful DAVK formation, a 3 h incubation of 70 mg lysine (K) with 1 g DAV was found optimal, since after the reaction, only a few milligrams of unreacted lysine remained (Fig. 3). Details of DAVK optimisation is included in the Supplementary Fig. S3, S4 and S5. The favourable zeta potential of DAVK thus obtained was − 25 mV at pH 8.0, 60 V, and 25 °C for efficient phosphate interaction. DAVK formation was confirmed using FT-IR analysis (Fig. 2c), which revealed stable product formation with peaks of amino acid groups of lysine derivative.

Optimisation of PDP encapsulation with DAVK was required for maximum phosphate interaction by DAVK (the outer surface of the encapsulation material) and removal by PDP (the inner core of encapsulation material) from synthetic media under simulated intestinal juice conditions. Therefore, maximal phosphate removal was used as the target response for the optimisation process. Important parameters affecting the DAVK encapsulation process on PDP were amounts of DAVK, PDP, and incubation time. Temperature (37 °C) was kept constant for the optimisation of PDP encapsulation, since this temperature condition was pre-optimised for DAVK formation and stability and CFU counts of PDP and its bio-functionality were found to be unaffected at this temperature condition. DAVK (1 g) was kept constant as starting or reference material to start the optimisation process.

Using a one-factor-at-a-time approach, for 1 g of DAVK, different PDP levels were analysed for synbiotic formation up to 6 h and then each prepared synbiotic at different PDP levels evaluated for phosphate removal in vitro where the prepared synbiotic at optimal level of PDP (0.75 g) could remove a maximum of 10.58 mg (67.4%, w/w) of phosphate from the synthetic media (Table 1a). Similarly, after determining the optimal PDP level, the optimal incubation time (3 h) was determined at which the formulated synbiotic could remove a maximum of 10.61 mg of phosphate, in vitro (Table 1b).

**Table 1:** Phosphate removal capacity of DAVK-encapsulated PDP was analysed under simulated in vitro conditions containing synthetic media (initially, 15.70 mg phosphate used in the experiment). Data are represented by the mean with standard errors (SE). (a) Effect of different PDP levels for 1 g of DAVK for phosphate removal from in vitro broth; (b) effect of incubation time in PDP encapsulation with DAVK for phosphate removal from in vitro broth.

Ratio of PDP (g) to DAVK, 1 g	Phosphate content (mg) removal from in vitro broth	SE
(**a**)		
1.00:1	5.36	2.1
0.90:1	6.03	1.1
0.80:1	7.84^a^	1.3
0.75:1	10.58^b^	1.1
0.70:1	7.78^c^	1.0
0.60:1	7.36	1.2
0.50:1	3.09	1.3
0.40:1	1.36	1.2
0.30:1	1.03	1.4

Incubation time (h)	Phosphate content (mg) removal from in vitro broth	SE
(**b**)		
2	6.86^a^	0.4
3	10.61^b^	0.2
4	10.59^b^	0.3
5	10.56	0.2
6	10.58	0.1

*DAV* deacetylated *Aloe vera*; *DAVK* lysine derivative (K) of DAV; *PAO* phosphate accumulating organism; *PDP* phosphate deficient PAO.

Superscript symbols: a, b and c represent significant differences (*p* = 0.008, 0.006, and 0.003, respectively, by two-tailed ANOVA, with n = 3 and alpha value of 0.95).

Under optimised conditions, PDP encapsulation with/and DAVK synthesis for synbiotic formulation occurred simultaneously where 2 g DAV (wet wt.), 140 mg lysine, 1.5 g PDP (wet wt.), and 24 mg sucrose mixed and incubated at 37 °C for 3 h. The formulated synbiotics could remove 10.66 mg of phosphate (67.89%) from the in vitro broth containing synthetic media as phosphate source (initially, 15.7 mg of phosphate).

**Figure 4:**
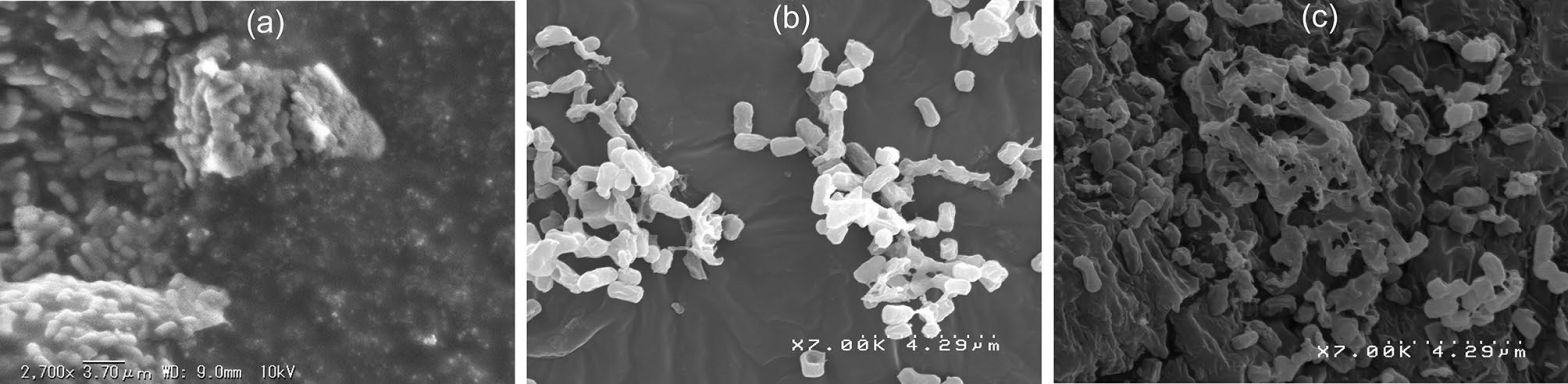
Scanning electron microscopy (SEM) analysis of *Lactobacillus casei* JCM1134 as phosphate-deficient cells (PDP) and under stepwise encapsulation conditions. (**a**) PDP, (**b**) lysine (K) derivative of deacetylated *A. vera* (DAVK)-encapsulated PDP, and (**c**) spray-dried synbiotic formulation (SP).

After successful optimization of synbiotic formulation, morphological and structural analyses of PDP and DAVK encapsulated PDP were performed by scanning electron microscopy (SEM) (Fig. 4). As shown in Fig. 4a, PDP was observed at high concentration where each cell was clearly visible with good structural evidence. In contrast, cells are found bound together and covered with layers of some sticky substance, certainly DAVK in Fig. 4b. We observed that the particle size of PDP (0.902 μm) increased to 1.503 μm following PDP encapsulation (Table 2).

**Table 2:** Monitoring of parameters for synbiotic components, spray drying, and at each step of in vitro experiments using synthetic media as phosphate source (initial phosphate content, 15.70 mg).

Parameter	Synbiotic components		Spray drying		In vitro broth with synthetic media		
	DAV	PDP	Before	After	Saliva	Gastric juice	Intestinal juice
Particle size (μm)	–	0.902	1.503	1.214	1.528	1.519	1.521
Sugar (mg/mg)	0.986	–	0.545	0.782	0.583	0.571	0.569
Zeta mV (pH)	− 39.17 (8.0)	–	− 25.01 (8.0)	− 0.94 (7.0)	–	–	− 25.26 (7.8)
Remaining phosphate in vitro (mg)	14.70 (PDP + DAV)^a^	15.45	–	–	15.70	15.68	5.04

Standard errors, < 8%; *p* < 0.05 by two-tailed ANOVA with alpha value of 0.95.

*DAV* deacetylated *Aloe vera*; *PAO* phosphate accumulating organism; *PDP* phosphate deficient PAO.

^a^The mixture of PDP and DAV removed 1 mg phosphate/mL, and the remaining phosphate content is mentioned in the table.

– not applicable.

### SP formation and characterisation

For maximum phosphate removal under in vitro conditions, synbiotic formulation (DAVK encapsulated PDP) must successfully pass the saliva and gastric environment and enter in the simulated intestinal phase of the in vitro experiment in good biologically working state. Good working state of synbiotics means that the maximum colony forming units (CFU) of PDP in the intestinal phase must be obtained, which can be achieved by the formation of a protective layer on synbiotics (DAVK encapsulated PDP) to pass the saliva and gastric environment successfully and safely. Therefore, different percentages of DAVK-encapsulated PDP (synbiotic) were spray-dried with a supernatant of DAV centrifugation (sacrificial outer layer to protect PDP from simulated saliva and gastric juice) and analysed for the CFU counts of formulated SP after passing the simulated saliva and gastric juices. The SP obtained at 0.5%, 1%, 2%, 4%, and 6% (DAVK-encapsulated PDP/DAV supernatant; w/v) showed 1.2 × 10^4^, 1.1 × 10^4^, 2.4 × 10^3^, 3.6 × 10^2^, and 3.1 × 10^2^ CFU/25 mg SP, respectively, after passing simulated saliva and gastric juice conditions. Since the CFU of SP prepared using 1% and 0.5% (w/v) DAVK-encapsulated PDP/DAV supernatant had a similar outcome under the tested in vitro conditions, 1% (w/v) DAVK-encapsulated PDP/DAV supernatant was used for further studies. The SP yield from spray drying was 75% (w/w) from a 200-mL suspension.

The standardised conditions for SP formulation are summarised in Table 3a. The successful formulation of second layer on SP was confirmed via SEM, particle size, zeta potential, and CFU counts (in vitro). Compared to Fig. 4b, the SEM image of SP (Fig. 4c) showed more substances around and between the cells in surplus quantity, confirming that the DAVK encapsulated PDP is enveloped with the substances present in the spray drying medium, which was the DAV supernatant. The moisture content in SP was found to be approximately 6.38% (w/w) (Table 3b). SP showed 1.1 × 10^4^ CFU/25 mg of SP with a sugar content of 0.782 mg/mg, as shown in Table 3b. The surface properties of SP were observed using the zeta potential (− 0.94 mV at pH 7.0, 60 V), particle size (1.214 μm), colour measuring system (light pink, L* = 93.31, a* = 0.05, and b* = 7.32) and are shown in Table 3b. The higher sugar content of SP was attributed to the encapsulation of *A. vera* carbohydrates with a relatively higher zeta potential. However, the size of SP was smaller than that of DAVK-encapsulated PDP owing to the elimination of water upon spray drying, which was also confirmed through particle size analysis (Table 2). These results supported the second layer formation on synbiotic.

**Table 3:** SP formulation under standardized conditions and characterization.

Standard conditions for SP formulations	
(**a**)	
DAV	2 g
PDP	1.5 g
Lysine	140 mg
Sucrose	24 mg
Temperature	37 °C
Incubation time	3 h
Spray drying medium (T_i_, 80 °C; T_o_, 60 °C; 100 mL/h feed rate)	Prepared synbiotic in supernatant of DAV at 1% (w/v)

SP characterization	
(**b**)	
Moisture content (% w/w)	6.38
CFU/25 mg SP	1.1 $\times$ 10^4^
Particle size (µm)	1.214
Sugar content (mg/mg)	0.782
Zeta potential (mV, pH 7.0, 60 V)	− 0.94
Colour (L*, a*, b*; 93.31, 0.05, 7.32)	Light pink
Phosphate removed from in vitro broth containing synthetic media (mg)	10.66

### Evaluation and comparison of SP

The phosphate removal capability of SP was compared to the synbiotic formulation using DAV and PDP. Formulations were added at 100 mg/mL. The SP formulation could remove 10.66 mg phosphate under in vitro conditions, which was significantly higher than that achieved by DAV and PDP formulations, which removed 1.0 and 0.25 mg phosphate, respectively (Table 2) with statistical significance of *p* < 0.05. This difference in the phosphate removal capability demonstrates the necessity of DAVK synthesis and its encapsulation as an inner layer over PDP.

The increase in SP size observed in the simulated saliva was due to water retention, its decrease in simulated gastric juice was owing to hydrolysis of the sacrificial (outer) layer, whereas it remained constant under simulated intestinal conditions (Table 2). Similarly, the hydrolysis of the sacrificial layer implies the reduction of the sugar content of SP under simulated saliva and gastric conditions; however, the later sugar content of SP is maintained under simulated intestinal conditions. The SP integrity under saliva and gastric juice, and subsequently the successful phosphate removal performance by SP under in vitro conditions, demonstrates the need of a second layer on synbiotic for protection from saliva and gastric juice.

**Figure 5:**
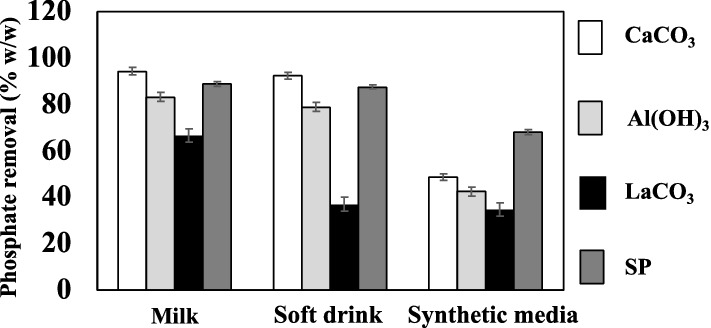
Comparison of spray-dried synbiotic formulation with commercial phosphate binders. Phosphate-rich foods that were used included milk, a soft drink, and synthetic media. Significant *p*-values (*p* < 0.05 by two-tailed ANOVA, with n = 3 and alpha value of 0.95) were obtained and standard errors are shown as error bars.

The phosphate removal capacity of SP was compared with that of commercial phosphate binders in phosphate-rich sources, such as milk, soft drink, and synthetic media, which contained 0.314, 1.413, and 15.7 mg phosphate /mL, respectively. Next to CaCO_3_, SP was the best phosphate accumulator at removing phosphate with 89% and 87% (w/v) from milk and soft drink, respectively (Fig. 5), whereas Al(OH)_3_ and LaCO_3_ showed lower phosphate removal capacities. In synthetic media, SP was the best phosphate accumulator (67% [w/v] phosphate removal), whereas CaCO_3_, Al(OH)_3_, and LaCO_3_ exhibited second-, third-, and fourth-best phosphate removal capacities, respectively (Fig. 5). In our opinion, this ambiguous result between SP and CaCO_3_ performance for phosphate removal from milk, soft drink, and synthetic media was due to the different accessible forms of phosphate present in different phosphate-rich sources; SP exhibited different interactions with them. Altogether, the inference of these results remains the same.

## Discussion

Probiotics and prebiotics are known to remove uremic toxins such as urea, uric acid, cresol, *p*-cresyl sulphate, and trimethylamine-*N*-oxide (TMAO) from the gut to prevent kidney damage. In a double-blind study, 40 g of arabinoxylan oligosaccharides (a prebiotic) was reported to reduce TMAO, indoxyl sulphate, *p*-cresyl glucuronide, *p*-cresyl sulphate, and urea in 40 participants^31^. Ranganathan et al. conducted a pilot study of 46 participants and evaluated the successful reduction in serum creatinine, uric acid, and blood urea nitrogen by the administration of Kibow Biotics (probiotics: *Streptococcus thermophilus* KB 19, *L. acidophilus* KB 27, and *B. longum* KB 31) at 9 × 10^10^ CFU/day^32^. Another study comprising 33 participants in a double-blind experiment of probiotics use (9 × 10^10^ CFU/day: *S. thermophilus*, *L. acidophilus*, and *B. longum*) showed successful results for reducing serum urea, indoxyl sulphate, and *p*-cresyl sulphate^33^. Few synbiotic studies have also reported to prevent CKD by lowering the uremic toxins in the gut. Probinul neutro, composed by inulin (prebiotics; 6.6 g/day) and probiotics (*L. plantarum*, *L. casei subsp. rhamnosus, L. gasseri*, *B. infantis, B. longum*, *L. acidophilus*, *L. salivarius, L. sporogenes, S. thermophilus;* 5.7 × 10^10^ CFU/day) was successfully tested for the reduction of plasma p-cresol in a double-blind study of 30 haemodialysis patients^34^. Similarly, Familact (probiotics: *L. casei, L. acidophilus, L. bulgaricus, L. rhamnosus, B. breve, B. longum, S. thermophilus*; and prebiotics: fructo-oligosaccharides) is documented for the alleviation of blood urea nitrogen, serum creatinine, and uric acid in a double-blind study of 66 patients with stage three and four of CKD^35^.

*A. vera* was also assessed for the prevention of kidney damage in some reports. The short-chain fatty acids (SCFAs: acetic, lactic, and propionic acids) produced during the fermentation of *A. vera* by *L. fermentum* showed the recovery response in CKD animal and human models. SCFAs are well reported for their health benefits, such as decline of renal fibrosis, amelioration of tubular damage, decreasing serum creatinine and blood urea nitrogen, to improve the renal function^36^. Another study revealed that aluminium toxicity can result in renal damage and elevated levels of creatinine, urea, and uric acids; however, these toxic levels can be reduced by treatment with *A. vera*^37^. One more interesting study conducted by Tanomand et al*.* on adult mice with multiple sclerosis concluded that the alcoholic extract of *A. vera* gel can reduce urea, uric acid, and creatinine levels and tissue modification of the kidney in such patients^38^.

Prior to this study, no investigation has reported any prebiotic or probiotic holding the potential to remove phosphate from the gut and prevent hyperphosphatemia (except us). Herein, we aimed to provide a healthier approach for phosphate removal by evaluating a SP formulation composed of PDP and *A. vera*, in which *A. vera* encapsulated layers protected the PDP allowing it to function as a phosphate accumulator under simulated in vitro conditions. *A. vera* is reported as a good encapsulating agent to prevent damages and preserve the core biomolecule integrity and functionality^39^. In a recent review, *A. vera* gel was considered as a good encapsulating material since it is edible, safe, and biodegradable. It provides enough restrictions for moisture and gas exchanges to conserve the firmness, colour, and flavour of the fruits and vegetables. Additionally, the antimicrobial and antioxidative properties of *A. vera* minimises the microbial growth of the encapsulated product^40^.

Furthermore, microbial encapsulation can be achieved through various materials for different purposes. For example, alginate encapsulation of *B. bifidum*, *L. acidophilus* BCRC 10695, and *L. casei* NCDC 298 is widely incorporated in functional food formulations^41–43^. Chitosan-coated alginate beads were used to encapsulate *L. casei* 01 and *L. acidophilus* 547 to prepare yoghurt^44^. Furthermore, modification of cellulose with positively charged diethylamino ethyl groups better encapsulated *L. plantarum* 8RA-3 than did neutral and negatively charged cellulose^45^. In our study, application of a prebiotic as encapsulating agent for the target probiotic imparts prebiotic efficacies, which is more advantageous and cost-effective than the application of conventional encapsulating materials. Herein, SP formulation was customised in terms of the formation of inner and outer layers, which are crucial and specific to achieve optimal phosphate removal by PDP under the harsh environment of the in vitro broth. If either layer or the modification of components is missing, it will be difficult to obtain fully biologically functional SP in the in vitro experiments (Table 2).

In this study, the conventional method of preparing synbiotics by mixing probiotics and prebiotics did not work out well. The in vitro results showed that the application of either elite PAO alone or synbiotic formulation using elite PAO and DAV was not sufficient for effective phosphate removal, owing to the lack of PAO stability and inefficient phosphate affinity or interaction. Therefore, strategic formulation and SP tailoring are essential to safeguard the elite PAO and its bio-functionality through double encapsulation of *A. vera*, in which the supernatant of the DAV centrifugation formed the outer layer to protect PDP from saliva and gastric juices, while DAVK formed the inner layer to interact with and remove phosphate from the in vitro broth. To achieve efficient encapsulation and maintain SP integrity under the in vitro conditions, the sugar moiety of *A. vera* was changed from hydrophilic to hydrophobic by a deacetylation reaction. The unfavourable zeta potential of the obtained DAV was − 39.17 mV, which was modified by lysine, forming DAVK with a favourable zeta potential of − 25 mV for the removal of phosphate from the in vitro broth. Moreover, the reduced SP size and sugar content confirmed the role of the sacrificial (outer) layer for the intact SP delivery, since the final zeta potential of SP under simulated intestinal conditions was similar to that of DAVK-encapsulated PDP (that is SP with inner layer) before spray drying. Good working conditions of SP under simulated intestinal conditions are shown in Table 2.

To the best of our knowledge, this is the first report of DAVK synthesis and strategic formulation of SP under optimised conditions, which provided novel avenues to derivatise oligomers and tailor synbiotic formulations for targeted responses, respectively. Although there are few reports on acemannan modification and its therapeutic applications, a novel study described the use of acemannan modification as an antibiotic vehicle in which surface-modified lipid nanoparticles were synthesised with chitosan-conjugated acemannan, an acetylated polymannose of aloe gel isolate, to deliver rifampicin intracellularly^46^. In another study, a hydrophilic gel was prepared from acemannan and *Moringa oleifera* and introduced as a layer between the bone and dental implant to enhance the ossification process^47^. *A. vera* gel is reported to be modified with chitosan and used in the regenerative therapies as a vehicle gel to deliver mesenchymal stem cells to second-grade burn injuries for therapeutic healing purposes^48^.

After safe SP delivery to the simulated intestinal condition, the DAVK (inner layer) of SP had a high zeta potential, possibly by attracting negatively charged molecules including phosphates, which might facilitate phosphate transport to PDP and their accumulation, as indicated by the higher phosphate removal performance of SP with DAVK compared with DAV (Table 2); this result signifies DAV derivatization with lysine. Stepwise assessment of phosphate content in the in vitro broth confirmed that phosphate accumulation by SP began in the simulated intestinal (duodenum, jejunum, and ileum) juice and accumulated 10.66 mg phosphate (Table 2) with 67.89% efficiency while maintaining SP integrity. Most phosphate removal by SP was achieved in simulated intestinal juice; this result coincides with another physiological report^19^.

Compared with commercial binders, SP and CaCO_3_ exhibited comparative performance on phosphate removal. However, the applicability of CaCO_3_ is restricted to patients with a positive calcium balance due to potential hypercalcaemia development and vascular and extraosseous calcification^49^. Although the developed SP can be applied to prevent hyperphosphatemia in patients with hypercalcaemia and renal disease, the calcium chelation ability of *L. casei* and the applicability of SP should also be considered. To prevent hyperphosphataemia, SP is a healthier and promising phosphate accumulator, since lactic acid bacteria are safe (Generally Regarded As Safe, GRAS) and commonly utilised in functional foods. Moreover, the consumption of lactic acid bacteria does not require medical approval. Furthermore, *A. vera* is industrially popular for its therapeutic applications. This is the first study to conduct the successful in vitro trial of phosphate-removing potential of SP from phosphate-rich sources and performed well with the most studied phosphate binders.

This tailored functional food formulation would benefit the medicinal or functional food industries to prevent hyperphosphataemia, and DAVK synthesis may advance the applications of *A. vera* in diverse areas. The technique developed to synthesise DAVK can be useful to produce novel aminoglycans and their potential application can be explored for health benefits. The developed strategic encapsulation technology can also be applied for the protection of heat and pH-labile potential molecules with possible surface modifications of the encapsulating material. This targeted encapsulation strategy can be utilised for the production of other functional food formulations and drug delivery platforms at specific sites with good bio-functionality.

The results from SP and CaCO_3_ with synthetic media and phosphate-rich food (Fig. 5) suggest that different phosphate forms under in vitro conditions have different affinities for CaCO_3_ and SP. This novel interpretation of the diet is a critical factor for evaluating and prescribing the best-suited phosphate binders, since the phosphate removal performance depends on its affinity to accessible phosphate.

A limitation of this work included the scaling-up of the SP formulation, as the spray drying and DAV production showed limited yields. We found that the yield of the SP after spray drying was significantly reduced on the scale-up, probably due to the high levels of hygroscopic sugar content in *A. vera.* This is a challenge for the development of the SP, which must be addressed through the detailed study on suitable wall material. Similarly, low DAV yields were obtained, but those could be improved with the modification and/or optimization of the deacetylation reaction of *A. vera.* Both the issues of low DAV and spray drying yields must be addressed by conducting appropriate scale-up studies for the commercial production of SP.

In future studies, we intend to incorporate SP in different food systems to advance the currently available functional foods and potentially enhance the quality of life of patients with CKD. In addition, animal experiments are obligatory for the commercialisation of the SP formulation in the prevention of hyperphosphatemia.

## Methods

### Chemicals and equipment

De Man, Rogosa, and Sharpe (MRS) media was purchased from BD Biosciences (Franklin Lakes, NJ, USA). *A. vera* powder was obtained from Morinaga Milk Industry Co. (Tokyo, Japan). ⍺-amylase, pepsin, and bile salt were purchased from Fujifilm Wako Pure Chemical Corporation (Osaka, Japan), and pancreatin was obtained from Sigma (St. Louis, MO, USA). A commercial soft drink and milk (pasteurised) were purchased from a local market. All chemical reagents used in this study were of analytical grade.

Data were collected using a muffle furnace (MFP-300A; IKEDA Scientific Co., Tokyo, Japan), an organic elemental analyser (UNICUBE; Elementar Analysensysteme GmbH Langenselbold, Germany), a scanning electron microscope (JSM-6330F; Tokyo, Japan ), a FT-IR spectrometer (FT-IR 300; JASCO, Tokyo, Japan), a zeta potential instrument (Melles Griot, Carlsbad, CA, USA), an Isoton II diluent instrument with particle size data analyser (Muiltisizer 4, Beckman Coulter, Brea, CA, USA), a high-performance liquid chromatograph (LC-10AS, Shimadzu, Kyoto, Japan), a gas chromatograph (GC-4000, TC-1; GL Sciences, Tokyo, Japan), and a colour measuring instrument (Konica Minolta, Tokyo, Japan).

Samples were prepared using a lyophiliser (Eyela, Tokyo, Japan) and spray drier (pilot-scale SD 1000; Eyela) for further analysis. Double distilled water was used in all experiments.

### Sub-culture of PAO and preparation of phosphate-deficient cells

*L. casei* JCM1134 as a PAO was inoculated from 1-mL frozen MRS broth stocks (diluted twice with 40% (v/v) glycerol) into 200-mL Erlenmeyer flasks containing 100 mL sterilised MRS medium, and grown for 24 h at 37 °C. PDP was prepared by incubating active PAO in filter-sterilised transformation media at pH 6.0 for 8 h^18^. Cells were then obtained by centrifugation at 1,915 × *g* for 5 min.

### DAVK preparation

#### DAV preparation

Deacetylation of *A. vera* was conducted with some modifications^50^. *A. vera* powder at 5% (w/v) was dissolved in distilled water, pH was adjusted to 8.0 using 1 M NaOH, and incubated in a water bath at 65 °C for 12 min. After cooling, the suspension was centrifuged at 5,320 × *g* for 10 min and the pellet (1.5 g, wet weight) was collected. The deacetylation reaction, zeta potential, and sugar content of freeze-dried DAV were analysed using an FT-IR spectrometer, a zeta potential analyser, and the phenol sulphuric acid method^51^, respectively.

#### Aloe vera and DAV characterisation

Moisture content and elemental analyses of *A. vera* were performed (Supplementary Table S1). Sugar components of *A. vera* and DAV preparations were determined (Supplementary Table S2, Supplementary Fig. S1 and S2).

#### Lysine derivatisation of DAV and its fabrication on PDP

Under optimised conditions (detailed optimisation data are presented in supplementary Fig. S3, S4 and S5), DAV (1 g) and lysine (70 mg) were mixed and incubated at 37 °C for 3 h. Samples were collected every 30 min and centrifuged at 1,915 × *g* for 5 min. The supernatant was analysed for free lysine using amino acid estimation^52^. DAVK formation was confirmed by FT-IR and zeta potential analysis.

DAVK encapsulation on PDP or synbiotic formulation was optimised (one factor at a time) for maximum phosphate removal efficiency (target response) under simulated intestinal juice using synthetic media (15.7 mg/mL) as a phosphate source. Different PDP levels were mixed with DAVK (1 g) and incubated at 37 °C for 6 h. Samples were then evaluated for their phosphate removal capacity. Under optimal conditions of DAVK encapsulation on PDP, optimisation of incubation time was achieved by varying the incubation time from 2, 3, 4, 5, and 6 h and samples were evaluated for their phosphate removal capacity. Encapsulation of DAVK on PDP was confirmed by SEM, zeta potential, sugar content and particle size analysis.

### SP formulation

Using a spray drying method, 0.5%, 1%, 2%, 4%, and 6% (w/v) of the DAVK-encapsulated PDP or synbiotic were mixed with supernatant of DAV centrifugation to prepare the SP powder, with maximum CFU (target response) counted under simulated saliva and gastric juice. To obtain a stable SP powder, minimise the water activity, and avoid SP spoilage, spray drying was performed at an inlet temperature of 80 °C and outlet temperature of 60 °C, with a 100 mL/h feed rate. The phosphate removal capacity of SP (formulated using DAVK) was compared with SP (formulated using DAV) and PDP.

### SP characterisation

SP was analysed for CFU count and sugar content^51^. CFU per 25 mg SP were estimated by spreading a diluted SP suspension on sterilised MRS agar and counting the colonies after incubation at 37 °C for 24 h.

#### Moisture content

SP powder (500 mg) was weighed in a crucible muffle and kept in a muffle furnace at 550 °C for 5 h. This step was repeated until a constant weight of ash was obtained. The moisture content was estimated by subtracting the initial weight of SP powder with ash weight.

#### Scanning electron micrographs

Air-dried samples were spread on carbon double-sided black tape (Nisshin EM Co., Tokyo, Japan) and mounted for platinum sputtering (E-1030 Ion sputter, HITACHI, Tokyo, Japan) for 250 s, 6 Pa vacuum, and 15 mA current. Platinum-coated (25 nm) samples were analysed by SEM.

#### Zeta potential determination

Samples were diluted to 0.02% (w/v) in 50 mM sodium citrate–phosphate buffer at pH 7.0 or 8.0, 60 V, and 25 °C. The Smoluchowski equation was used to determine the zeta potential^53^.

#### Colour measurement

SP powder (4000 mg) was mounted on a disc and analysed in a colour measuring instrument^54^. The colour was expressed using the L*, a*, and b* system.

#### Particle size measurement

Pellets of centrifuged samples were diluted to 1% (w/v) in distilled water. A 1 mL suspension was added to Isoton II diluent, the mixture was placed inside a Beckman Coulter counter, and the size distribution of the suspension was determined by the number of particles using the Multi 4e version 1–2 software (Multisizer Coulter Counter, Beckman Coulter).

### Evaluation of SP with commercials under in vitro conditions

The composition of the synthetic media is shown in Supplementary file (note). As per previous reports, the recommendation of low phosphorous daily consumption for renal patients should be of 1,000 mg, whereas the normal phosphorous daily consumption should be of 1,500 mg^55^. Synthetic media as phosphate rich source was prepared by adding 1,000 mg phosphate salts in 100 mL. However, the in vitro broth (4 mL, synthetic media: saliva: gastric juice: intestinal juice [1:1:1:1]) comprised known and complex and natural compounds that added up to the total phosphate content (15.7 mg in 4 mL of broth) of the in vitro broth containing digested synthetic media. Thus, it corresponded to the normal phosphorous daily consumption ($\sim$1,500 mg).

Milk, soft drink, and synthetic media were used for in vitro analysis at 25% (v/v). SP, CaCO_3_, LaCO_3_, and Al(OH)_3_ were added to the in vitro samples at 100 mg/mL as phosphate removal agents. Then, each agent was analysed for its phosphate removal capacity from each source.

**Figure 6:**
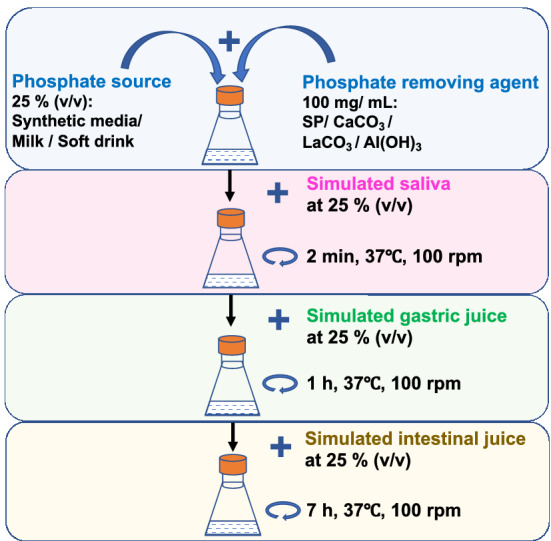
A schematic diagram of a gastrointestinal simulation system for the evaluation of different phosphate removing agents from the in vitro broth, which contains phosphate-rich sources.

A gastrointestinal simulation system was developed as previously described, with some modifications^56^, shown in a schematic diagram in Fig. 6. A phosphate source and phosphate removal agent were added to 1 mL sterilised human saliva containing filter-sterilised ⍺-amylase (1%, w/v). The mixture was incubated at 37 °C for 2 min. Then, 1 mL sterilised gastric juice was added and incubated at 37 °C for 1 h, and 1 mL sterilised intestinal juice was added and incubated at 37 °C for additional 7 h. The composition of the simulated saliva, gastric, and intestinal juices is shown in Supplementary Fig. S3.

SP samples were collected at each step of the in vitro experiment and centrifuged at 1,915 × *g* for 5 min. The supernatant was analysed to determine the remaining phosphate content, while the SP pellet was analysed for sugar content, zeta potential, and particle size. Samples of other phosphate removal agents were collected at the end of the experiment and analysed for the remaining phosphate content.

### Analytical methods

#### Measurement of phosphate using the ascorbic acid method

Phosphate content was assessed using the ascorbic acid method as described previously^57^, with some modifications. The collected samples were diluted and hydrolysed for 30 min at 121 °C using potassium peroxodisulphuric acid (0.25 g) and 5 N sulphuric acid (0.54 mL). Samples were then filtered through 0.2-µm filters and assessed for phosphoric acid by mixing 4.2 mL filtered samples with 0.8 mL of a 10:3:6:1 mixture of 5 N sulphuric acid, K_2_(SbO)_2_C_8_H_4_O_10_·3H_2_O (0.1372 g/100 mL), (NH_4_)_6_Mo_7_O_24_·4H_2_O (4.0 g/100 mL), ascorbic acid (1.32 g/75 mL), formic acid (0.6 g) in 0.4954 mL of EDTA (25 mg). Phosphoric acid was used as the standard and the obtained standard Eq. (1) with R^2^ = 0.9941. Reactions were assayed at 880 nm after 10 min incubation at 25$\sim$30 °C.

1 $$y = 0.5734 \times x + 0.0316$$

#### Functional group determination

Freeze-dried DAV and DAVK samples (3 mg) were ground with 100 mg KBr and placed in a FT-IR spectrometer for spectral determination at a wave number range of 400$\sim$4,000 cm^−1^.

### Statistical analysis

All experiments were performed in triplicate (n = 3). Two-way ANOVA was performed for multiple comparisons between data sets using MS Excel 2016 (version 15.26) to determine the significant differences (*p* < 0.05). Error bars (standard error of the mean) were included for all numerical data.

## Supplementary Information

Supplementary Information.

## Data Availability

All the required data and material are included in the article.
