# Author Correction: Tailored synbiotic powder (functional food) to prevent hyperphosphataemia (kidney disorder)

**DOI:** 10.1038/s41598-021-98194-3

**Published:** 2021-09-13

**Authors:** Ajeeta Anand, Shigeki Yoshida, Hideki Aoyagi

**Affiliations:** Faculty of Life and Environmental Sciences, University of Tsukuba, Tsukuba, Ibaraki 305‑8572 Japan

Correction to: *Scientific Reports* 10.1038/s41598-021-95176-3, published online 13 August 2021

The original version of this Article contained errors in Figure 5, where the x-axis label “Soft drink” was incorrectly given as “Coca cola”.

The original Figure 5 and accompanying legend appear below.

**Figure 5:**
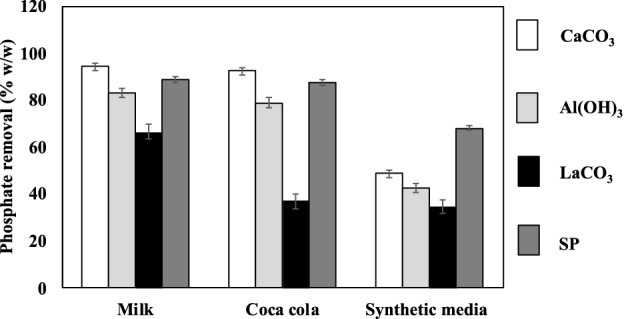
Comparison of spray-dried synbiotic formulation with commercial phosphate binders. Phosphate-rich foods that were used included milk, a soft drink, and synthetic media. Significant *p*-values (*p* < 0.05 by two-tailed ANOVA, with n = 3 and alpha value of 0.95) were obtained and standard errors are shown as error bars.

The original Article has been corrected.

