# Supplementary material for: Tailored synbiotic powder (functional food) to prevent hyperphosphataemia (kidney disorder)
Source: Sci Rep. 2021 Aug 13;11:16485. doi: 10.1038/s41598-021-95176-3 (PMC8363651; doi:10.1038/s41598-021-95176-3)
Supplement: Supplementary file 1 — Supplementary Information. [file 41598_2021_95176_MOESM1_ESM.pdf]

Tailored synbiotic powder (functional food) to prevent hyperphosphataemia (kidney disorder)

Ajeeta Anand, Shigeki Yoshida and Hideki Aoyagi*

Faculty of Life and EnvironmentalSciences, University of Tsukuba, Tsukuba, Ibaraki 305-8572, Japan.

Corresponding author: Hideki Aoyagi, Faculty of Life and EnvironmentalSciences, University of Tsukuba, Tsukuba, Ibaraki 305-8572, Japan.

E-mail: aoyagi.hideki.ge@u.tsukuba.ac.jp

Supplementary data:

Characterization of *Aloe vera* and its products of deacetylation reaction

Moisture content and elemental analysis of *Aloe vera* powder

Methods

Moisture content:

A half gram of *Aloe vera* powder was weighed in a crucible muffle and kept in muffle furnace at 550ºC for 5 h. This step was repeated several times until we obtained a constant weight of ash. The moisture content was found to be 71 % (w/w).

Elemental analysis:

Elemental analysis of *Aloe vera* was conducted by the research facility centre for science and technology (University of Tsukuba, Japan) using Elementar (UNICUBE) and the results are displayed in Supplementary Table S1.

Results

Supplementary Table S1. Elemental analysis of *Aloe vera* powder.

**Elements**	**Weight percent**
**C**	30.62
**H**	5.01
**N**	0.27
**S**	0.58

Sugar content in *Aloe vera*

The sugar content of *Aloe vera* was analysed using three methods: An Anthrone assay, glucose assay kit, and gas chromatography (GC), where samples were prepared using sulphuric and trifluoroacetic acid (TFA) at different conditions.

Methods

Hydrolysis methods:

Two moles TFA was used to hydrolyse *Aloe vera* at 121ºC for 1 h. While sulphuric acid was used at two molarities (0.5 and 1.2 M) to hydrolyse *Aloe vera* at 100ºC for 2 h. Samples were neutralised before further study.

Sugar assay:

Diluted *Aloe vera* (300 mg) was mixed with 1 mL of 0.2% (w/v) anthrone. Sulphuric acid was heated in boiling water bath for 5 min, cooled, and the absorbance read at 620 nm[1]. While a standard glucose estimation protocol was followed as per the Glucose assay kit (glucose CII-test, Japan).

Gas chromatography

Sample preparation:

Hydrolysate samples (5 mL) were neutralised using $BaCO_3$ and 1 mL of 4 mg/mL methyl beta-glucoside (internal standard). Neutralised samples were filtered through filter Advantec No. 5C and the permeate was reduced with $NaBH_4$ at pH 8.0 and 30ºC for 1.5 h. Amberlite resin (IR 200C) was used to convert $NaBH_4$ to $NaBO_3$ and later evaporated with methanol using a rotary vacuum evaporator. A dried sample were acetylated with 1 mL of acetic anhydride and pyridine 30ºC overnight, and later dried with a rotary vacuum evaporator. The dried sample was mixed was mixed with 0.5 mL dichloromethane and further analysed using GC.

Analysis condition:

GC: GC 4000 (GL science), TC-1

Mobile phase: Nitrogen gas

Injection temperature: > 50 ºC

Detector temperature: 250 ºC

Injection volume: 1 µL

Packed column: Silicon PPE -6 ring

Detector: FID

Results:

Supplementary Table S2. Sugar components analysis of *Aloe vera* using various analytical methods.

**Analytical method**	**Sulphuric acid hydrolysate**	**TFA hydrolysate**
	Glucose/Mannose % (Hydrolysis acid molarity)	
GC	14.3 / 6.1 (0.5 M)	5.6 / 2.8 (2 M)
GC	10.5/ 4.2 (1.2 M)	
**Assay**	**Sugar %** (Hydrolysis acid molarity)	
Anthrone	65.5 (0.5 M), 46.5 (1.2 M)	40 (2 M)
Glucose kit		50

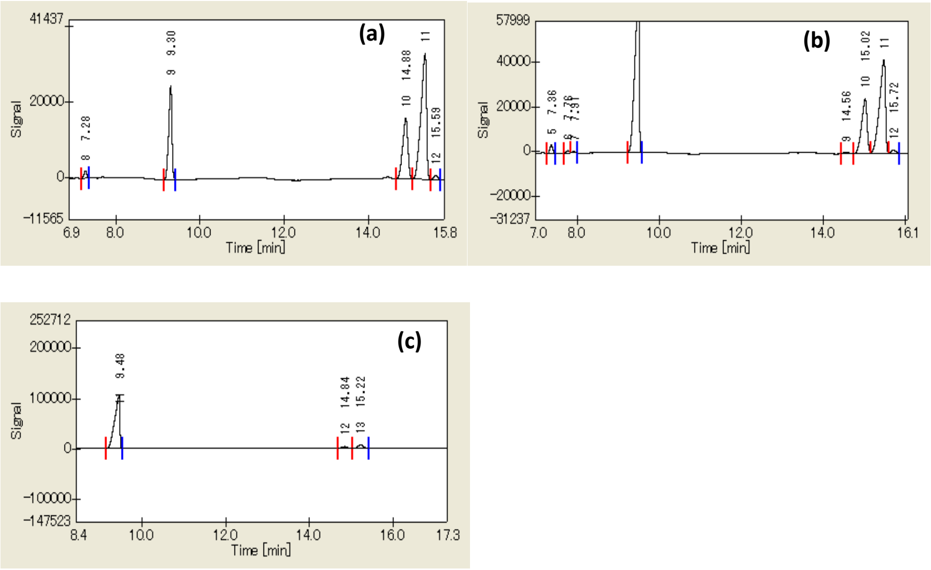

Supplementary Figure S1. GC analysis of *Aloe vera* carbohydrate under various acid hydrolysis: (a) 0.5 M H_2SO_4, (b) 1.2 M H_2SO_4, and (c) 2 M TFA.

We observed different sugar content under different acid treatment and assay procedures as shown in Supplementary Table S2. Although sugar content obtained from sulphuric acid (1.2 M) and the TFA hydrolysis, using anthrone assay, were similar; sugars components differed by double. With low molarity of sulphuric acid, sugar content increased to 65 % (w/w) but higher molarity (2 M) of TFA (weaker acid) had less sugar content.

As per GC analysis depicted in Supplementary Table S2 and Supplementary Fig. S1, sugar in *Aloe vera* is composed of glucomannan where the amount of glucose is almost twice that of mannose. Still, a glucose enzymatic assay resulted in 50% (w/w) glucose in the TFA hydrolysed sample. Therefore, the exact content of sugar and its components remains unclear. However, sugar in *Aloe vera* is composed of glucose and mannose in 2:1 ratio.

Molecular weight determination of deacetylated *Aloe vera* (DAV) and its supernatant

Experimental design:

HPLC: LC-10AS, LC.

Mobile phase: 50 mM Sodium phosphate buffer + 150 mM NaCl + 0.05% NaN_3 (pH 6.8)

Flow rate: 0.25 mL/min

Column: TSK gel G4000PWxL (Pmax 2.0) + G3000PWxL (Pmax 4.0) (7.8 mm x 30 cm)

Injection: 100 µL

Detector: RID

Dextran standards

T 2000

T 500

T 70

T 40

T 20

Sucrose

Sample preparation:

Samples of DAV and its supernatant obtained using the deacetylation reaction of *Aloe vera* were freeze-dried and prepared at 0.5%, pH 6.0 in water and filtered (pore size, 0.02 µm).

Freeze drying of samples were performed at shelf temperature of -10 °C, vacuum at 15 Pa and trap temperature at -50 °C.

Results:

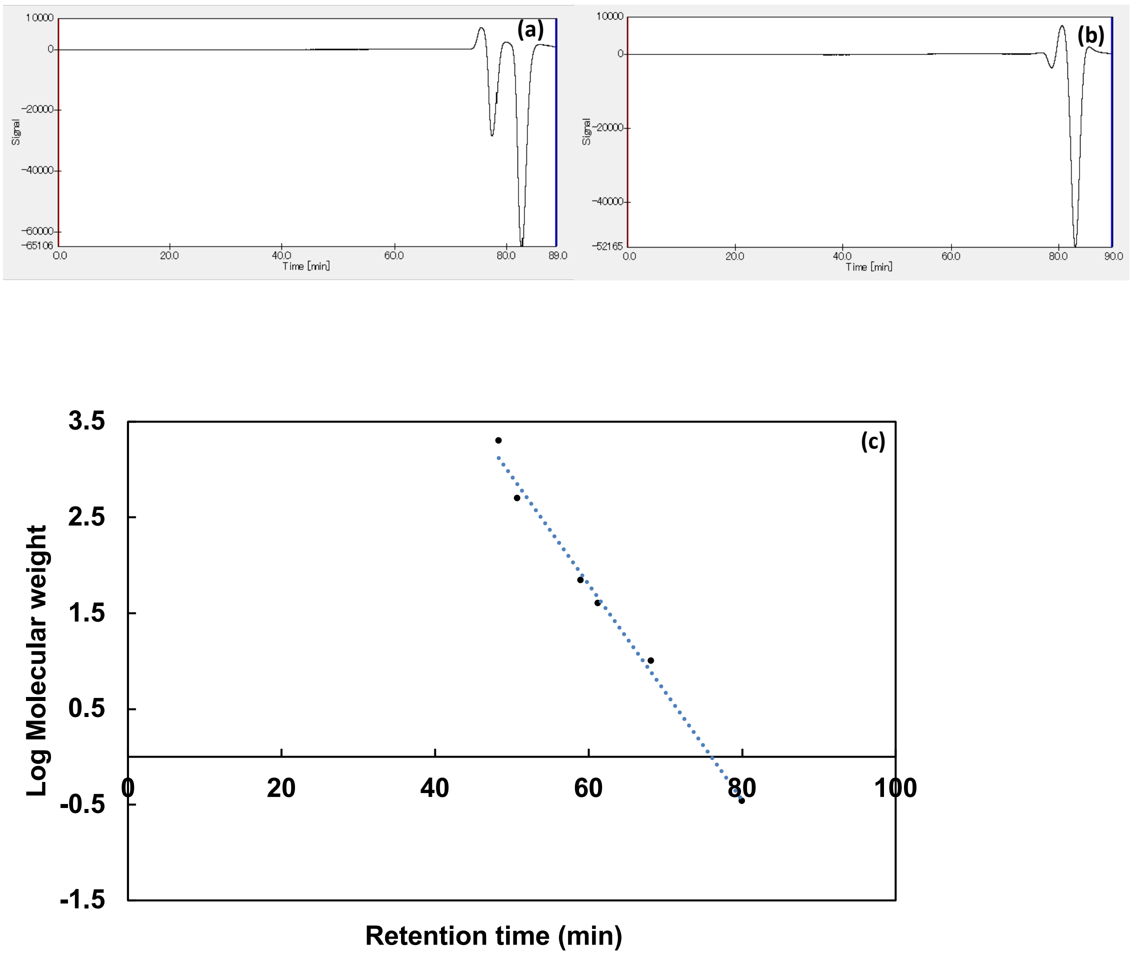

Supplementary Figure S2. HPLC analysis of DAV preparation. (a): Peak of DAV in HPLC chromatogram, (b) peak of DAV supernatant in HPLC chromatogram at pH 6.0, and (c) calibration curve of dextran standards and sucrose.

A peak of DAV eluted at 75.5 min (Supplementary Fig. S2) was the only peak detected in the chromatogram. The peak of DAV corresponds to the average molecular weight of oligosaccharides of approximately 1.13 KDa consisting of six to seven monomers. While the peak of DAV supernatant eluted at 76.1 min corresponds to 968.7 Da (Supplementary Fig. S2). A calibration curve was drawn for the molecular weight analysis of *Aloe vera* and its deacetylation products using sucrose, as shown in Supplementary Fig. S2.

DAV was prepared according to Chokboribal *et al*[2] with modifications. The authors

reported that the polysaccharides in *Aloe vera* ranged between 190 to 220 KDa and composed of mannose (57%), glucose (22%), and galactose (17%); while deacetylated polysaccharides in *Aloe vera* was of 165-185 KDa. The results of the present study differed from that of previous findings.

One factor at a time optimization for deacetylated *Aloe vera*-lysine (K) DAVK formation

DAV and lysine (K) were reacted at pH 8.0 and 37°C and formed lysine derivatized DAV or DAVK which was confirmed with FTIR analysis. DAVK formation is important for the competitive removal of phosphate from *in vitro* broth. For optimization, our target was to obtain maximum DAVK per g of DAV. Therefore, the level of DAV was kept constant (1 g). For the stability of DAV under simulated intestinal juice, pH 8.0 is important; therefore pH 8.0 was also kept constant during optimization process. The reaction temperature was kept 37°C as constant because later, PDP need to be encapsulated with DAVK at optimal temperature for the maximum PDP counts (very important for the phosphate removal performance by SP under *in vitro* broth) that is 37°C; additionally, at this temperature, no amino acids were degraded.

Experiment for DAVK optimization

For DAVK formation, there were two significant parameters that needed to be optimized: levels of lysine (K) and reaction time. One factor at a time approach was first applied for the different K levels: 50 mg, 100 mg, 200 mg, 400 mg, 600 mg, and 800 mg; and the other reaction parameters were 1 g DAV, pH 8.0 and volume was adjusted to 1 mL using double distilled water and vortexed, then kept at 37°C for 6 h. Samples were collected after 6 h and centrifuged at 1,915 × *g* for 5 min. The supernatant was analysed for free lysine using amino acid estimation[3].

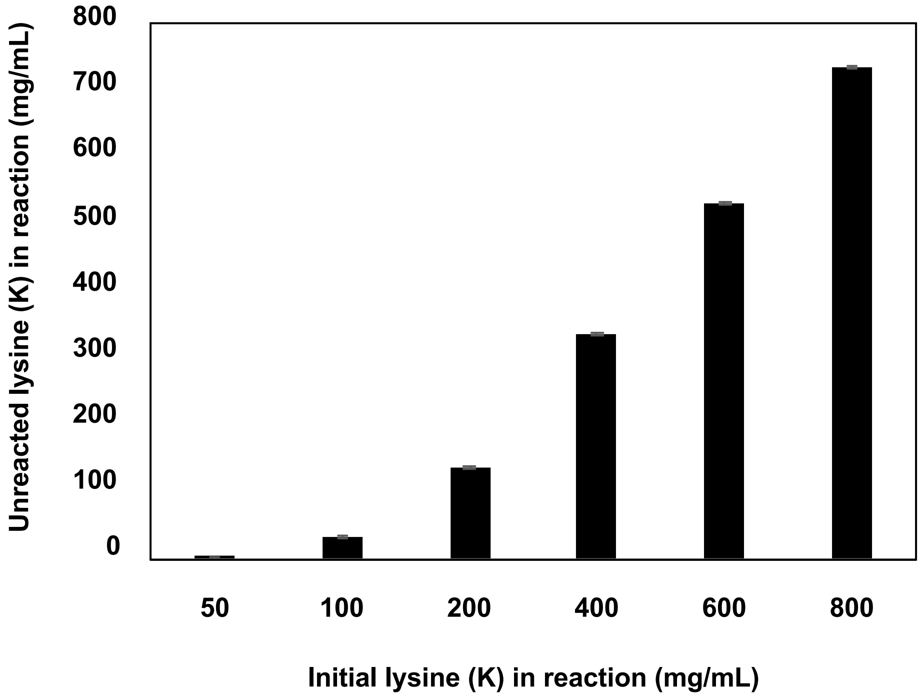

Supplementary Figure S3. Result of preliminary experiment for the lysine (K) levels determination to maximize DAVK formation per g of DAV.

As shown in Supplementary Fig. S3, among the different levels of lysine, 50 to 100 mg of lysine in reaction was found enough to completely react with 1 g of DAV as in the supernatant of reaction, the unreacted lysine concentrations were found the least. Then, we repeated the same experiment with lysine levels at 50, 60, 70, 80 and 90 mg/mL.

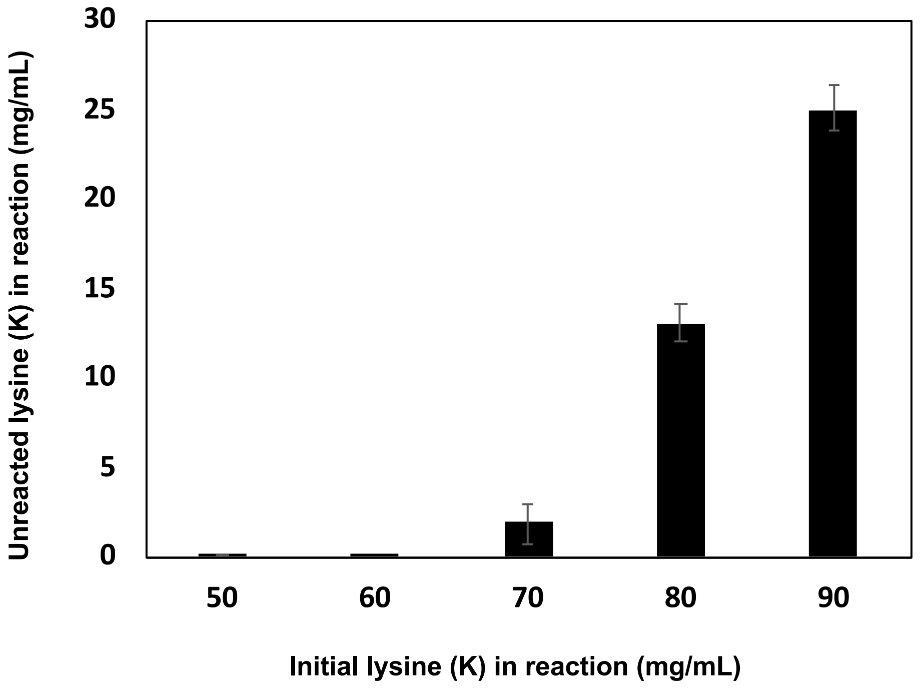

Supplementary Figure S4. Determination of optimal lysine (K) level to maximize DAVK formation per g of DAV.

As shown in Supplementary Fig. S4, 50 and 60 mg/mL of lysine were completely reacted with 1 g of DAV, while 80 and 90 mg/mL of lysine were found excessive. However, 70 mg/mL of lysine was found appropriate with insignificant amount of unreacted lysine that shows the enough amount of lysine was used to react with 1 g of DAV completely. Therefore, 70 mg/mL of lysine was found optimal for the optimization of DAVK formation from 1 g of DAV.

Reaction time was optimized for the maximization of DAVK formation by analysing the unreacted lysine in reaction mixture. The unreacted lysine levels were evaluated at different reaction time (1, 2, 3, 4, 5 and 6 h) under the following reaction conditions: 1 g DAV and 70 mg lysine were mixed, and volume was adjusted to 1 mL using double distilled water, then

vortexed at pH 8.0, and kept at 37°C. Samples were collected atdifferent time intervals and analysed for unreacted lysine.

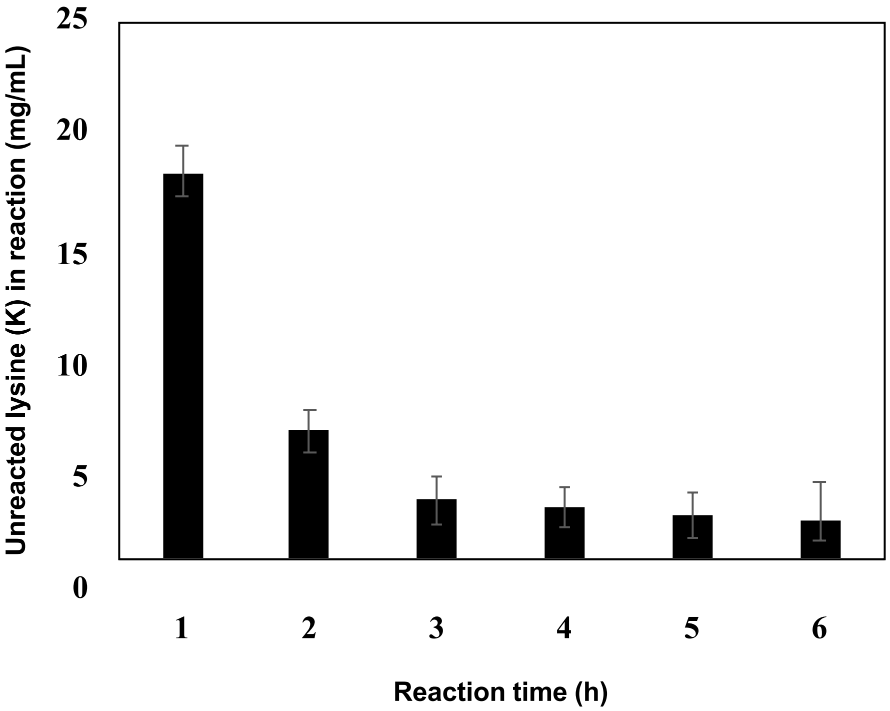

Supplementary Figure S5. Determination of optimal reaction time (h) level to maximize DAVK formation per g of DAV.

As shown in Supplementary Fig. S5, the significance levels of reaction time data at different time intervals merged at and beyond 3 h. This result concluded the three hour of reaction time was suitable since further time elongation did not result in significant data or lower unreacted lysine in reaction mixture.

The optimized parameters of DAVK formation were as follows: 1 g DAV and 70 mg lysine were mixed, and volume was adjusted to 1 mL using double distilled water, then vortexed at pH 8.0, and kept at 37°C for 3 h of reaction time.

Supplementary Note:

In vitro simulations

- **SP or other phosphate binder:** 100 mg/mL
- **Synthetic media** (% [w/v]): Glucose (1), sucrose (1), fructose (1), lactose (1), Na_2HPO_4(0.7), KH_2PO_4 (0.3), $NaNO_3$ (0.2), and tryptone (2).
- **Saliva:** Human saliva + *a*-amylase (1 % [w/v]): **2 min**
- **Gastric juice** (% [w/v]): NaCl (0.62), KCl (0.22), $CaCl_2$ (0.02), $NaHCO_3$ (0.12), and pepsin(0.3) at pH 3.0: **1 h**
- **Intestinal juice** (% [w/v]): $NaHCO_3$ (0.64), KCl (0.023), NaCl (0.13), bile salt (0.3), and pancreatin (0.1) at pH 7.4: **7 h**

1. Dische, Z. Color reactions of carbohydrates. In: Methods in carbohydrate chemistry, Vol. 1 (Whistler, R. L. & Wolfrom, M. L. (eds)). Academic Press, New York and London, 478-512 (1962).

2. Chokboribal, J., Tachaboonyakiat, W., Sangvanich, P., Ruangpornvisuti, V., Jettanacheawchankit, S. & Thunyakitpisal, P. Deacetylation affects the physical properties and bioactivity of acemannan, an extracted polysaccharide from *Aloe vera*. *Carbohydr Polym* **133**, 556–566 (2015). doi: 10.1016/j.carbpol.2015.07.039

3. Hwang, M. N. & Ederer, G. M. Rapid hippurate hydrolysis method for presumptive identification of group *B. streptococci*. *J. Clin. Microbiol.* **1**, 114–5 (1975).
